# The Association between Stressful Events and Food Insecurity: Cross-Sectional Evidence from Australia

**DOI:** 10.3390/ijerph15112333

**Published:** 2018-10-23

**Authors:** Jeromey B. Temple

**Affiliations:** Demography and Ageing Unit, Melbourne School of Population and Global Health, University of Melbourne, Melbourne 3010, Australia; Jeromey.Temple@unimelb.edu.au; Tel.: +61-3-9035-9900

**Keywords:** food insecurity, stressors, stressful life events, access to food, food equality

## Abstract

A considerable body of empirical evidence exists on the demographic and socio-economic correlates of food insecurity in Australia. An important omission from recent studies, however, is an understanding of the role of stressful life events, or stressors in explaining exposure to food insecurity. Using nationally representative data from the 2014 General Social Survey and multivariable logistic regression, this paper reports on the association between 18 discrete stressors and the likelihood of reporting food insecurity in Australia. The results, adjusted for known correlates of food insecurity and complex survey design, show that exposure to stressors significantly increased the likelihood of experiencing food insecurity. Importantly, stressors related to employment and health approximately doubled the odds of experiencing food insecurity. The results underscore the complex correlates of food insecurity and indicates that conceptually it interacts with many important social and economic problems in contemporary Australia. There is no simple fix to food insecurity and solutions require co-ordination across a range of social and economic policies.

## 1. Introduction

Food insecurity is the “limited or uncertain availability of nutritionally adequate and safe foods or limited or uncertain ability to acquire acceptable foods in socially acceptable ways” [1]. In 1975, Australia ratified the United Nations International Covenant on Economic, Social and Cultural Rights, recognising the fundamental human right for its citizenry to be free from hunger [2]. More recently, in 2015, Australia further ratified the United Nations 2030 Agenda for Sustainable Development and the Sustainable Development Goals which seek to eliminate poverty and inequality, with a target of ‘zero hunger’ by 2030 [3].

Indeed, it is now widely understood that food insecurity is a problem facing not only low and middle-income countries, but also high-income countries such as the USA, Canada and Australia [4,5]. In Australia, approximately 4–5% of the population are estimated to be food insecure, due to a lack of financial resources, with about 40% of this group (or 2% of the population) going without food consequently [6,7]. However, the experience of food insecurity is not evenly spread throughout the Australian population, with a growing number of studies showing that constellations of socio-economic, demographic and geographic factors are associated with food insecurity. For example, young age, being divorced or separated, low income, low education, low financial resources, a high number of resident children, poor health, not owning your home, being unemployed, being an Aboriginal or Torres Strait Islander and measures of spatial disadvantage are all associated with experiencing food insecurity in Australia [7,8,9,10,11,12,13,14,15,16,17,18,19,20,21,22,23].

One important omission from recent studies, however, is an understanding of the role of stressful life events, or stressors, in explaining exposure to food insecurity. Stressors are events, whether anticipated or not, that can have a deleterious effect on the wellbeing of individuals and their families (e.g., onset of a serious health condition or unanticipated unemployment). Independent of known risk factors of food insecurity, an analysis of the association between stressors and food insecurity may provide evidence as to why some households beyond the bottom quintile of household income experience food insecurity in high income countries such as Australia.

International studies, mostly qualitative, have provided some evidence that stressors are associated with, or are a precursor to experiencing food insecurity. In two qualitative studies of low-income older Americans, major sickness and unexpected expenses and medical bills were key factors explaining food insecurity [24,25]. Moreover, family events such as Christmas were cited as a precursor to food insecurity, due to the financial costs associated with filial obligations such as gift giving [25]. A recent US mixed methods study provides some evidence that exposure to adverse childhood experiences (e.g., abuse, neglect, household instability) was associated with experiencing food insecurity later in adulthood [26]. A further qualitative American study found that stressful events such as those related to health and employment were related to food insecurity, but were also mediated by families ‘capabilities’ to offset negative consequences [27]. This finding is supported by a recent quantitative study which found that families adjusting to negative life events with low levels of income and social support were at a much greater risk of child hunger [28]. In Canada, both the onset of chronic disease and problem gambling were found to be associated with food insecurity in higher-income households [29].

Within the Australian literature, there have been few studies examining the link between stressors and food insecurity. Australian studies have, however, investigated the coping mechanisms used to avoid hunger when stressors such as homelessness, enduring social disadvantage and exogenous policy changes to welfare payments occur [20,30,31]. Generally, it is widely acknowledged that stressors may be an important determinant of food insecurity. For example, Burns (2004) has suggested “Although most persons living in poverty are at risk of food insecurity, it cannot be assumed that they are, in fact, food insecure. In addition, for many reasons, including factors such as ill health, disability, sudden job loss, and high living expenses, persons above the poverty line cannot be assumed to be food secure” [32], p. 7. Furthermore, in Temple’s (2008) study of food insecurity in Australia, it is noted “It may be that in times of sudden unemployment, divorce, death or unexpected illness, greater stress is placed on family resources. The ability to negotiate these stresses is likely to contribute to the prevalence of food insecurity” [7], p. 662.

In this study, nationally representative data were used to examine the association between stressors and food insecurity. Firstly, the likelihood of food insecure persons (relative to the food secure) reporting a stressor in the previous 12 months was examined. Secondly, the prevalence of food insecurity categorised by 18 discrete stressors was calculated. Finally, multivariable logistic regression models were used to examine the association between individual stressors and the odds of food insecurity, once extensive controls were accounted for.

## 2. Materials and Methods

### 2.1. Survey Data

Data used in this study were from the 2014 General Social Survey (GSS) conducted by the Australian Bureau of Statistics (ABS) between March and June 2014. Using a face-to-face interview along with prompt cards, the ABS collected information using a Computer Assisted Interviewing (CAI) questionnaire on a range of domains to understand the “multi-dimensional nature of relative advantage and disadvantage across the population, and to facilitate reporting on and monitoring of people's opportunities to participate fully in society” [33]. The GSS was designed to provide nationally and state representative estimates across these domains. From a sample of 18,574 private dwellings, 16,145 dwellings were used due to issues of scope or uninhabited dwellings. In total, 80% fully responded, yielding a sample of 12,932 people aged 15 years and over.

The GSS included persons who were usual residents of private dwellings at the time of the survey. This sampling design meant that several populations were excluded including those living in non-private dwellings (e.g., hostels, hospitals, short-stay caravan parks). Also excluded were diplomatic or defence personnel of overseas governments stationed in Australia, those whose usual place of residence was outside of Australia, or those living in very remote areas of discrete Aboriginal and Torres Strait Islander communities. People experiencing homelessness were also excluded from the survey.

### 2.2. Measurement

Two questions were included in the GSS instrument to identify exposure to stressors. Firstly, the interviewer asked: “Have any of these been a problem for you or anyone close to you, during the last 12 months?”. A prompt card (Card F15) was shown to respondents listing: 1. Serious illness, 2. Serious accident, 3. Death of a family member of close friend, 4. Mental illness, 5. Serious disability. Respondents were than further prompted, repeating the question with a second prompt card (Card F16) listing: 10. Divorce or separation, 11. Not able to get a job, 12. Involuntary loss of job, 13. Alcohol or drug related problems, 14. Witness to violence, 15. Abuse or violent crime, 16. Trouble with police, 17. Gambling problem, 18. Discrimination because of ethnic or cultural background 19. Discrimination for any other reason, 20. Bullying and/or harassment, 21. Removal of children, 22. Other. Using these questions, variables measuring 18 distinct stressors were generated.

Measurement of food insecurity in the GSS is included in the financial stress, resilience and exclusion module. Respondents were asked “In the last 12 months, have any of these happened because you were short of money?” A prompt card (Card K1) was shown to the respondent. Respondents who indicated that they went without meals due to a shortage of money were coded as being food insecure. The measurement of going without a meal due to a shortage of money is considered a measure of considerable financially attributable food insecurity, indicative of both inadequate food intake and food depletion [7].

### 2.3. Statistical Model

To examine the association between stressors and food insecurity multivariable logistic regression models were fitted. Using the raw logit coefficients, adjusted odds ratios (AOR) were calculated, which measure the change in the odds of experiencing food insecurity given an experience of each stressor, once all other factors in the model are controlled for. Regression models were estimated for each stressor independently. Given that food insecurity attributable to financial constraints is a relatively rare event, the stability of the logit coefficients were compared against those of a Scobit (Skewed Logit), Complementary Log-Log and Log Poisson regression model. The strength, significance and direction of parameter coefficients was highly comparable across all regression models, and the logit results are presented herein for simplicity.

Due to complex survey design, adjustments are necessary to generate correct variance estimates. The GSS includes 60 replicate weights on the data file to adjust for sample design and non-response. Utilizing an algorithm developed by Winter, the delete-one jackknife method was used to make the necessary replicate adjustments [34,35]. All analyses were conducted using Stata via the ABS Remote Access Data Laboratory.

#### Control Variables

Drawing upon previous Australian research outlined above, variables know to be associated with food insecurity were included in the regressions to control for potentially confounding effects. Specifically, the control variables included:

- Age: 15–29, 30–44, 45–59, 60+.
- Marital Status: Married, not married.
- Equivalised Household Income: The measure of household income available in the GSS is gross household income, adjusted or ‘equivalised’ using an equivalence scale to account for household size and placed in deciles. The ABS make this adjustment in household income to allow for welfare and financial wellbeing comparisons between households of different sizes and compositions. The categories included in the regression based upon income distribution include: 0 to 20%, 20% to 40%, 40% to 60%, 60% to 80%, 80% to 100%, not reported.
- Self-Rated Health: Poor or fair health, good or excellent health.
- Tenure: Renter, not a renter.
- University educated: Has university education, does not have university education.

Additional variables including gender and measures of geography were also included but were not found to be significant at the 95% critical level. For each stressor model, it would be inappropriate to include all control variables due to concerns regarding multicollinearity and other model misspecification issues. Specifically, for the divorce or separation model, marital status was omitted. For the illness, accident, mental illness and disability stressor models, self-rated health was omitted.

## 3. Results

### 3.1. Experiences of Stressors

Except for ‘other’ stressors, food insecure respondents were more likely to report experiencing each type of stressor, relative to the food secure (Table 1). Over one third of food insecure respondents reported not being able to get a job (40.5%), death of a family member or close friend (35.1%), mental illness (34.9%) and serious illness (33.3%). Large differences in the reporting of stressors between food insecure and secure respondents existed for not being able to get a job (40.5% v 16.8%) and mental illness (34.9% v 13.0%). Other considerable differences between food secure and secure respondents (with a difference in prevalence of greater than 10%) included bullying and harassment, alcohol or drug related problems, death of a family member or close friend, witness to violence, trouble with the police and serious illness. Whereas 38% of food secure persons reported no stressors in the last 12 months, only 14% of food insecure respondents did not experience stressors. In contrast, about half of the food insecure reported three or more stressors, compared with 16% of the food secure.

### 3.2. Prevalence of Food Insecurity by Stressor Type

Given that food insecure respondents were more likely to experience a range of stressors relative to their food secure peers, it is therefore not unexpected that the prevalence rates of food insecurity were much higher for those experiencing stressors (Table 2). Consistent with previous research using a similar measure, the prevalence of food insecurity with insufficient intake and food depletion was approximately 2% among the general population living in households [7]. The prevalence of food insecurity among those reporting no stressors in the previous 12 months was less than 1%. In strong contrast, the prevalence of food insecurity was very high among those reporting witness to violence (12.6%), removal of children (11.7%), abuse or violent crime (9.3%), trouble with the police (9.3%), discrimination—other reason (8.7%) and bullying or harassment (6.1%). Again, across all categories of stressors with the exception of ‘other’ stressors, prevalence rates of food insecurity were significantly above those who did not report any stressors or the general population level prevalence.

Although these descriptive results indicate significant differences in the prevalence of food insecurity by exposure to stressors, it is important to control for variables that may indicate spurious statistical relationships. For example, was the prevalence of food insecurity high among those reporting bullying or harassment due to a younger age profile of those reporting this stressor? Similarly, were prevalence rates of food insecurity among those reporting a mental health stressor high because of lower average levels of economic resources available to those with mental health conditions?

### 3.3. Regression Results

To control for confounding effects, multivariable logistic regression models were fitted to calculate odds ratios to measure the association between each stressor and food insecurity, once extensive controls for socio-economic factors previously shown to be associated with food insecurity in Australia were accounted for. Odds ratios adjusted for control variables (AOR) and unadjusted for control variables (UOR) are presented for transparency (Table 3). Comparing the adjusted and unadjusted results, the higher magnitude of the unadjusted odds ratios indicates the importance of the control factors in explaining food insecurity. This is further discussed below.

Broadly repeating the descriptive prevalence rates, those reporting witness to violence (AOR = 4.40 *p* < 0.001), removal of children (AOR = 4.58 *p* < 0.01), trouble with police (AOR = 3.70 *p* < 0.01), discrimination-other reason (AOR = 3.75 *p* < 0.01), abuse or violent crime (AOR = 3.26 *p* < 0.001) and bullying/harassment (AOR = 2.82 *p* < 0.001) were approximately three or more times more likely to report food insecurity.

Among the health-related stressors, mental illness (AOR = 2.87 *p* < 0.001), serious disability (AOR = 2.30 *p* < 0.01) and serious illness (AOR = 1.81 *p* < 0.01) approximately doubled the odds of experiencing food insecurity. Similarly, difficulties in the workplace also doubled the odds of experiencing food insecurity: not able to get a job (AOR = 2.49 *p* < 0.001) and involuntary job loss (AOR = 2.59 *p* < 0.001). An experience of a serious accident in the last 12 months was not associated with food insecurity (*p* > 0.05).

Table 4 displays results from a logistic regression model measuring the association between the number of stressors reported by the respondent and food insecurity. The full parameter coefficients measuring the relative role of the control variables are also included for context. The direction, magnitude and significance of the parameter coefficients for the control variables are highly comparable across all models in Table 3 and Table 4.

Findings from this analysis showed a slightly non-linear relationship between stressors and the odds of food insecurity. Relative to those reporting no stressors, those reporting one or two stressors were about 1.8 times more likely to be food insecure (OR = 1.76 *p* < 0.05). Those reporting three or four stressors were about 3.8 times more likely (OR = 3.75 *p* < 0.05) and those reporting five or more were approximately nine times more likely to report food insecurity (OR = 8.9 *p* < 0.05). As a proxy for the severity of stressors, these findings indicate that multiple stressors play a significant role in explaining exposure to food insecurity.

Contextualizing the results in Table 3 and Table 4, the control variables remain important determinants of food insecurity. Consistent with previous Australian studies, reporting food insecurity was about 60% less likely for university educated respondents (relative to those with no university education) and for those who were married (relative to the unmarried), OR = 0.39 *p* < 0.05 and OR = 0.39 *p* < 0.001, respectively. Reporting poor or fair self-rated health almost doubled the odds of experiencing food insecurity. Again, consistent with Australian studies, renters (as opposed to owners or purchasers of primary residences) were at a considerably greater risk of food insecurity in Australia (OR = 3.10 *p* < 0.05). As expected, household income (specifically equivalized household income) was strongly associated with food insecurity. Those in the top 20% of the income distribution were about 85% less likely to report food insecurity, relative to those in the bottom 20% of the distribution (OR = 0.15 *p* < 0.05).

The increased likelihood of experiencing food insecurity for those reporting multiple stressors raises the question of the composition of stressors experienced in the previous 12 months. Table 5 tabulates the types of stressors experienced by the number of stressors reported. Of those persons reporting 5 or more stressors, over half reported death of a family member or friend (59.8%), serious illness (65.9%), mental illness (60.5%), not able to get a job (67.5%) or bullying and/or harassment (51.7%). These percentages are considerably above those reported by people reporting only 1 or 2 stressors. For example, about 12% of those reporting 1–2 stressors report a mental illness shock, compared with 39% of those reporting 3–4 stressors and 61% of those reporting five or more stressors.

## 4. Discussion

Research in the fields of psychology and behavioural economics has emphasised the importance of stressors in explaining health and wellbeing throughout the life course [36,37]. Public health research too is increasingly recognising the important role that precariousness (through broader economic and political changes) plays in deleterious health and wellbeing outcomes [38]. Indeed, experiencing stressors and precariousness may be tied to experiences of economic and social inequality in Australia, contributing to overall food inequality [39]. Motivated by these broader frameworks, and by a limited number of American and Canadian qualitative studies, this study sought to examine the prevalence and association of 18 types of stressful events, or stressors, with food insecurity in Australia.

This analysis found that respondents reporting food insecurity were more likely to report stressors relative to food secure persons. Across 17 of the 18 stressor domains, food insecure people were significantly more likely to report experiences of a stressor. Moreover, the food insecure were significantly more likely to have encountered multiple stressors within the previous 12 months. Unsurprisingly then, the analysis herein demonstrated that the prevalence of food insecurity was considerably higher among those experiencing stressors. It was further demonstrated that once known determinants of food insecurity were controlled for, the odds of experiencing food insecurity remain highly statistically significant across 16 stressor types. Experiencing multiple stressors was also associated with significantly increased odds of food insecurity.

These findings raise the question of how stressors and precariousness can be built into policy or programs to address food insecurity? Of course, not all people who experience a stressor are at risk of food insecurity. Indeed, beyond individual levels of resilience and vulnerability, support systems from family, friends, the government and the broader community play an important role in managing the potential adverse effects of stressors [36]. However, in the absence of familial or other social-support mechanisms, how can Government support individuals at risk of food insecurity as they face potentially adverse stressors?

Solutions proposed for the broader community to protect financial wellbeing against stressors more generally include financial education, insurance and financial planning and preparedness [40]. However, for many food insecure people, lifelong disadvantage and detachment from the labour market makes such planning complex, if not unfeasible. The ability for policy to support people at risk of food insecurity also depends on the type of stressor. Of particular relevance, stressors related to both health (serious illness, mental illness, serious disability) and the labour market (not able to get a job, involuntary job loss) were strongly associated with food insecurity in this study.

Regarding labour market stressors, consideration should be given to the suitability of extant labour market programs for food insecure people. To assess this, it is necessary to firstly understand the barriers to labour force participation faced by food insecure people? Although much is known about labour market barriers more generally, there are no Australian studies that examine how policy can support unemployed food insecure people specifically. Moreover, it is well understood that higher levels of education are protective against experiences of both food insecurity and unemployment [8,9,41]. What are the barriers to education and training reported by food insecure people? These questions are being considered by the author in a subsequent analysis and underscore the complex policy solutions to food insecurity which must extend beyond food and nutrition programs alone. Relatedly, exogenous labour market shocks (e.g., unanticipated unemployment) raise the issue of the suitability of income support provided through the welfare support system. For example, the main income support payment available to unemployed people in Australia, the Newstart Allowance, has long been criticised for not providing a healthy living allowance, and the problem has compounded over time due to the method of indexation [42,43,44].

Onset of disability, mental health illness and other serious illness stressors were also strongly associated with food insecurity. The onset of health conditions, particularly multimorbidity and chronic conditions in Australia, has been shown to be associated with deleterious financial wellbeing [45,46]. For some Australians, analysis herein shows that health stressors may translate into a significantly higher likelihood of food insecurity. This however raises the question of the direction of the relationship between health and food insecurity. Is illness a precursor, an outcome or both with respect to food insecurity? For example, a recent scoping paper shows that a number of longitudinal studies find a bidirectional relationship between mental health and food insecurity [47]. Moreover, there is a significant literature on the detrimental mental health effects of unemployment [48]. Thus, there may be a complex relationship *between* and *within* stressors and food insecurity. Further research on the pathways between health and food security using longitudinal data is a priority. More generally, the particularly high odds ratios measuring the association between mental health stressors and food insecurity is also important given reported difficulties accessing and funding mental health care and support programs in Australia [49].

Among the strongest association between stressors and food insecurity identified in this study were for those related to violence and addiction including issues with alcohol or drug related problems and gambling. As noted previously, Canadian studies have noted gambling addiction issues as a possible precursor to food insecurity [29]. An Australian qualitative study of a charity-run soup kitchen noted issues of alcohol and illicit drug use and gambling in food insecure clients [50]. Issues of drug use (both licit and illicit) and gambling are important social problems in Australia with considerable implications for the economy as well as individual wellbeing [51,52]. The complexity of these problems and their solutions again underscore the multidimensional levers that must be employed by governments to address food insecurity. 

The association between removal of children and food insecurity was very strong and highly significant. Although this result is not unexpected, interpreting this result requires some caution due to the high prevalence of Aboriginal children in out-of-home care relative to non-Indigenous children [53]. As the Aboriginal and Torres Strait Islander population are at a considerably higher risk of food insecurity in Australia, it may be that the measure of removal of children is confounding this effect [23]. Notwithstanding, just under 3% of the Australian population were Aboriginal and Torres Strait Islanders in the 2016 Census of Population and Housing and it is not clear how many Aboriginal respondents were included in the GSS. Although Aboriginal and Torres Strait Islander people are included in the GSS, variables measuring Indigenous status are not available in the datafile. The National Aboriginal and Torres Strait Islander Social Survey (NATSISS) collects similar measures of food insecurity and stressors to those collected in the GSS and the analysis presented herein could be replicated for the Aboriginal and Torres Strait Islander population. 

As a proxy for the severity of stressors, reporting higher numbers of personal stressors was strongly associated with experiencing food insecurity. Descriptive statistics herein further illustrated that almost half of those reporting higher order stressors experienced death of a family member or friend, serious illness, mental illness, trouble finding employment and bullying or harassment. This raises the important point of the intercorrelation between stressors. For example, as noted above, the literature has highlighted the detrimental mental health effects of unemployment [48]. There is also a growing evidence base on experiences of violence by people living with a disability [54]. A further example of the intercorrelation between the various stressors is the relationship between onset of health conditions and difficulties finding or maintaining work and poverty [55,56]. The pathways between these stressors and food insecurity is an area that requires further research. Unfortunately, the GSS data are inappropriate to answer these questions for two reasons. First, the data are cross-sectional and retrospective questions were not asked on the timing of events. Second, the measurement of stressors in the GSS is aggregated so that it is not possible to identify whether the stressor was experienced by (i) the respondent; or (ii) somebody close to them. Detailed longitudinal data are required to disentangle these important questions for future research.

More generally, it is important to note that stressors as a risk factor for food insecurity should not lead to a disregard of other socio-economic factors and food supply characteristics placing individuals at risk. In their analysis of life events on family wellbeing, the Australian Institute of Family Studies (AIFS) notes “sole reliance on life events as indicators of the need for service provision would be unfortunate. The identification of individuals or families who are vulnerable to experiencing adverse events in the future is clearly important, but so too is the identification of families experiencing chronically destructive circumstances” [57]. In the context of food insecurity, the analyses herein should be interpreted as complementary to existing studies and further highlighting at risk population groups. This is further supported by the findings underscoring the relative importance of control variables, such as education, income and marital status, in explaining food insecurity.

### Study Strengths and Limitations

The key strength of this study is that it is the first Australian and one of a few internationally that have sought to examine the experiences of a range of stressors and their association with food insecurity. This addresses an important research gap in the extant quantitative literature on food insecurity. A further important strength of this study is that it is nationally representative. Booth and Smith’s (2001) key study bringing food insecurity to the fore for Australian dietitians and policy makers pointed to the key at risk populations of food insecurity in Australia [13]. Following this study, most Australian analyses of food insecurity tend to focus on population sub groups. For example, homeless or ‘at risk’ youth [58], students [10,59], refugees [60,61], children or families with young children [19,20,21], Aboriginal and Torres Strait Islanders [23,62], older Australians [14,16,22,23,63,64], those living in disadvantaged suburbs [20,65] or middle-income groups [11]. Most Australian studies also focus on cities or states: Adelaide and South Australia [9,58], Sydney and New South Wales [14,20,22,63] Brisbane Queensland [10,17,65] Melbourne and Victoria [11,15] or Tasmania and the Northern Territory [18,19]. There are very few Australian quantitative studies seeking a nationally representative view of the prevalence and correlates of food insecurity [7,16]. This study adds to that list.

Notwithstanding these strengths, there are several limitations of this study. Firstly, as the GSS data are cross-sectional, it is not possible to draw a causal link between stressors and food insecurity. Rather, the analyses show a clear association between the two, once known determinants of food insecurity have been controlled for. Second, and related to the above, due to the cross-sectional nature of the data, it is not possible to measure the complex pathways between the various stressors and food insecurity. It may be that some stressors are a precursor or outcome (or both) of food insecurity. More generally, food insecurity has been shown to be a cyclical phenomenon, varying over time. Longitudinal data are required to measure these complex pathways. A third limitation of this study relates to measurement of the experience of stressors. The GSS instrument asks whether the stressor impacted the individual respondent or someone close to them. The argument that stressors experienced by someone close to you would impact on your likelihood of food insecurity can clearly be made. For example, a spouse losing their job, or a respondent’s child becoming seriously ill. Furthermore, qualitative studies provide evidence of how shocks to one person’s health can impact on the food insecurity of all household members [66]. However, it may be that when the stressor is experienced by the respondent alone, the effect on the likelihood of experiencing food insecurity is stronger. Unfortunately, the GSS does not enable this disaggregation. However, this analysis would be possible for the Aboriginal and Torres Strait Islander population using NATSISS.

Furthermore, there are a range of exogenous events, such as natural disasters, that may impact exposure to food insecurity and are not measured in the GSS. This study has focussed on personal stressors only, but clearly natural disasters, even in a high-income context, will impact levels of food insecurity. For example, a recent American study found that, even after a recovery phase following Hurricane Katrina, almost one in four people reported food insecurity five years later [67]. Moreover, the personal stressors measured in the GSS exclude potentially positive life events, for example birth of a child or marriage. It may be that for some demographic groups positive life events reduce the likelihood of food insecurity, whereas for other groups it may increase exposure to food insecurity. For example, for some vulnerable populations, positive events such as birth of a child may place greater stress on family resources, leading to a higher likelihood of food insecurity. These data are currently unavailable in the GSS, and this presents an important area for future research.

A fourth limitation of this study is the measurement of food insecurity itself. Going without a meal due to financial constraints is considered a measure of considerable financially attributable food insecurity, indicative of both inadequate intake and food depletion [7]. However, food insecurity exists in circumstances beyond financial considerations alone. Indeed, a number of recent Australian studies have sought to pilot or test more comprehensive measures of food insecurity which include non-financial barriers to food [8,22,68]. These more comprehensive measures show that the prevalence of food insecurity is much higher than when measured based on financial restrictions in accessing food alone. How stressors impact non-financial forms of food insecurity is a priority for future research.

## 5. Conclusions

Noting these limitations and extensions, to the author’s knowledge, this is one of only a few studies to examine the association of a wide range of stressors with food insecurity. Analysis herein showed specific as well as multiple occurrences of stressful events or stressors were associated with food insecurity, independent of known risk factors. These results underscore the complex determinants of food insecurity in Australia and complement existing studies which heretofore have focussed on socio-economic and demographic correlates. Further confirmation of these findings with longitudinal data is a priority, in order to establish the complex pathways in and out of food insecurity and the role of stressors as either precursors or outcomes (or indeed whether a bidirectional relationship exists). Moreover, extending this study to the Aboriginal and Torres Strait Islander population and with more comprehensive measures of food insecurity could provide further insight into stressful events and food insecurity.

Designing policy interventions to support people at risk of food insecurity is key to reducing food insecurity in Australia. Unfortunately, the results from this study suggest that addressing food insecurity is not a straightforward task for policy makers. Many of the stressors interact with important and difficult social problems in Australia, for which there are no straightforward solutions. With further longitudinal research on the pathways within and between stressors and food insecurity, appropriate interventions for those at risk of particularly deleterious stressors, could be designed in tandem with nutrition programs. By addressing food insecurity alongside the related social and economic problems identified in this study, health and economic outcomes for vulnerable populations may be improved and inequalities in health and wellbeing addressed consequently. This approach views food security as a fundamental human right, as recognised by the Australian Governments agreement with key UN accords.

## Figures and Tables

**Table 1:** Stressors reported by Food Insecurity Status, Weighted (%), 2014.

	Food Insecure (%)	Food Secure (%)		n ^1^
**Type of Stressor** ^2^				
Divorce or Separation	17.6	11.3	***	1467
Death of Family Member/Close Friend	35.1	21.4	***	2957
Serious Illness	33.3	22.3	***	3008
Serious Accident	7.7	4.5	**	612
Alcohol or Drug Related Problems	20.7	6.8	***	988
Mental Illness	34.9	13.0	***	1769
Serious Disability	13.1	6.0	***	914
Not Able to Get a Job	40.5	16.8	***	1952
Involuntary Job Loss Witness to Violence	16.5	7.0	***	859
	15.8	2.2	***	426
Abuse or Violent Crime	12.6	2.5	***	428
Trouble with the Police	14.2	2.8	***	413
Gambling Problem	6.6	2.6	***	308
Discrimination-Ethnic or Cultural Background	5.5	2.1	***	301
Discrimination-Other Reason	7.6	1.6	***	262
Bullying and/or Harassment	20.6	6.4	***	929
Removal of Children	4.8	<1%	***	141
Other	<1%	<1%		88
**Number of Stressors** ^3^				
None	14.0	38.1	***	4845
1	21.1	30.2	***	3807
2	15.7	15.4		1991
3	15.9	7.4	***	1011
4+	33.4	8.9	***	1278
Total	100	100		
**Unweighted (n)**	403	12,529		12,932

^1^ Unweighted sample size per stressor; ^2^ Experiencing each stressor in the previous 12 months; ^3^ Number of stressors reported in previous 12 months. *** *p* < 0.001, ** *p* < 0.01. Significance tests denote test of proportion of exposure to each stressor by food insecurity status.

**Table 2:** Prevalence of food insecurity by stressor type, 2014.

	Weighted ^1^ (%)	Unweighted ^2^ (%)	
**Type of Stressor** ^3^			
Divorce or Separation	3.1	5.7	***
Death of Family Member/Close Friend	3.2	4.9	***
Serious Illness	2.9	5.1	***
Serious Accident	3.4	5.9	**
Alcohol or Drug Related Problems	5.8	9.7	***
Mental Illness	5.2	8.7	***
Serious Disability	4.2	7.9	***
Not Able to Get a Job	4.6	7.7	***
Involuntary Job Loss Witness to Violence	4.5	7.3	***
	12.6	16.4	***
Abuse or Violent Crime	9.3	15.0	***
Trouble with the Police	9.3	12.6	***
Gambling Problem	5.0	9.1	***
Discrimination-Ethnic or Cultural Background	5.1	9.0	***
Discrimination-Other Reason	8.7	14.1	***
Bullying and/or Harassment	6.1	9.0	***
Removal of Children	11.7	15.6	***
Other	2.0	5.7	
**Number of Stressors** ^4^			
None	<1%	<1%	-
1	1.4	1.7	**
2	2.0	3.9	***
3	4.2	6.2	***
4+	7.1	11.7	***
**Full Sample**	2.0	3.1	***

^1^ Prevalence weighted using ABS survey weights; ^2^ Unweighted prevalence; ^3^ Tests of proportions for type of stressor relative to not experiencing each stressor; ^4^ Tests of proportions for number of stressors relative to those reporting no stressors. ‘None’ is the comparison category; *** *p* < 0.001, ** *p* < 0.01, + *p* < 0.1.

**Table 3:** Odds ratios from multivariable logistic regression models of food insecurity, 2014.

	Odds Ratio (UOR) ^1^	Odds Ratio (AOR) ^2^	
**Stressor Type Models ^3^**			
Divorce or Separation	1.68	1.53	*
Death of Family Member/Close Friend	1.99	2.01	***
Serious Illness	1.74	1.81	**
Serious Accident	1.78	1.55	
Alcohol or Drug Related Problems	3.58	2.35	***
Mental Illness	3.59	2.87	***
Serious Disability	2.36	2.30	**
Not Able to Get a Job	3.35	2.49	***
Involuntary Job Loss Witness to Violence	2.62	2.59	***
	8.27	4.40	***
Abuse or Violent Crime	5.67	3.26	***
Trouble with the Police	5.75	3.70	***
Gambling Problem	2.69	2.53	*
Discrimination-Ethnic or Cultural Background	2.76	2.17	*
Discrimination-Other Reason	5.04	3.75	***
Bullying and/or Harassment	3.79	2.82	***
Removal of Children	6.79	4.58	**
Other	1.00	0.66	

^1^ Unadjusted Odds Ratios with no control variables included. ^2^ Odds Ratios adjusted for controls including age, marital status, household income, self-rated health, housing tenure and education. ^3^ Multivariable logistic regression models estimated for each stressor; *** *p* < 0.001, ** *p* < 0.01, * *p* < 0.05; Standard errors calculated using survey replicate weights.

**Table 4:** Multivariable logistic regression model of number of stressors and food insecurity, 2014.

	Odds Ratio (AOR) ^1^	
**Number of Stressors ^2^**		
0	-	
1–2	1.76	*
3–4	3.75	***
5+	8.90	***
**Control Variables**		
Age		
15–29		
30–44	1.42	+
45–59	1.42	
60+	0.40	**
**University Education**		
Yes	0.39	*
Married		
Yes Tenure-Renting	0.39	***
	
Yes	3.10	***
Poor Self Rated Health		
Yes	2.28	***
**Equivalent Household Income**		
0–19%	-	
20–39%	0.81	
40–59%	0.52	*
60–79%	0.28	*
80–100%	0.15	**
Unknown	0.71	

^1^ Adjusted Odds Ratios (AOR) with controls for all variables included in the model. ^2^ Count of the number of stressors reported by the respondent in the previous 12 months; *** *p* < 0.001, ** *p* < 0.01, * *p* < 0.05, + *p* < 0.10; Estimates adjusted using survey replicate weights.

**Table 5:** Percentage of persons experiencing each stressor by number of stressors reported (%), 2014.

	Number of Stressors ^1^		
	1–2	3–4	5+
**Stressor Type (%) ^2^**			
Divorce or Separation	12.0	28.9	48.5
Death of Family Member/Close Friend	29.8	42.6	59.8
Serious Illness	29.0	50.3	65.9
Serious Accident	4.8	9.6	24.4
Alcohol or Drug Related Problems	4.1	23.8	46.3
Mental Illness	12.5	39.5	60.5
Serious Disability	6.2	16.5	26.4
Not Able to Get a Job	19.0	43.8	67.5
Involuntary Job Loss Witness to Violence	5.7	19.4	45.3
	0.7	5.8	28.9
Abuse or Violent Crime	1.0	8.3	24.3
Trouble with the Police	1.4	7.3	30.1
Gambling Problem	1.3	8.8	19.5
Discrimination-Ethnic/Cultural Background	1.0	6.1	18.9
Discrimination-Other Reason	<1%	4.9	17.9
Bullying and/or Harassment	4.2	18.0	51.7
Removal of Children	<1%	2.2	6.7
Other	<1%	<1%	1.4
Unweighted (n)	5804	1566	733

^1^ Number of stressors reported in previous 12 months. ^2^ Percentage of respondents in each category of stressor counts (1–2, 3–4, 5+) reporting each stressor.
